# Developing an Ontology Representing Fall Risk Management Domain Knowledge

**DOI:** 10.1007/s10916-024-02062-2

**Published:** 2024-04-25

**Authors:** Fatimah Altuhaifa, Dalal Al Tuhaifa

**Affiliations:** 1 School of Computing and Information Technology, University of Wollongong, Northfields Ave, Wollongong, NSW 2522 Australia; 2 Saudi Arabia Ministry of Higher Education, Riyadh, Saudi Arabia; 3 Microbiology laboratory department, Maternity and Children’s Hospital, Al Imam Ali Ibn Abi Talib St, Al Muraikabat, Dammam, 32253 Saudi Arabia

**Keywords:** Knowledge representation, Health informatics, Fall risk, Fall prevention, Information retrieve

## Abstract

Ontologies serve as comprehensive frameworks for organizing domain-specific knowledge, offering significant benefits for managing clinical data. This study presents the development of the Fall Risk Management Ontology (FRMO), designed to enhance clinical text mining, facilitate integration and interoperability between disparate data sources, and streamline clinical data analysis. By representing major entities within the fall risk management domain, the FRMO supports the unification of clinical language and decision-making processes, ultimately contributing to the prevention of falls among older adults. We used Ontology Web Language (OWL) to build the FRMO in Protégé. Of the seven steps of the Stanford approach, six steps were utilized in the development of the FRMO: (1) defining the domain and scope of the ontology, (2) reusing existing ontologies when possible, (3) enumerating ontology terms, (4) specifying the classes and their hierarchy, (5) defining the properties of the classes, and (6) defining the facets of the properties. We evaluated the FRMO using four main criteria: consistency, completeness, accuracy, and clarity. The developed ontology comprises 890 classes arranged in a hierarchical structure, including six top-level classes with a total of 43 object properties and 28 data properties. FRMO is the first comprehensively described semantic ontology for fall risk management. Healthcare providers can use the ontology as the basis of clinical decision technology for managing falls among older adults.

## Introduction

A fall is a sudden event in which a person falls to the floor, ground, or a lower level [1]. Falls are recognized as a significant public health issue and cause an estimated 684,000 deaths a year globally, making them the second most fatal type of unintentional injury [2]. Taking steps to reduce the rate of falls is extremely important to reduce the number of fall-related injuries and deaths [3]. Moreover, the financial, physical, and psychological costs of falls are reduced when they are predicted and prevented [4]. According to Health Direct Australia, most falls are preventable [5], primarily through risk assessment [6]. Knowledge of fall risk can be obtained from daily clinical records and official health websites like that of the World Health Organization (WHO). Since researchers have recently paid considerable attention to falls and injuries [7], useful information about falls can be extracted from these websites and daily clinical records. Software and computers can facilitate the exchange of information between researchers and the extraction of information about fall risk.

The challenge lies in leveraging this information effectively, necessitating the conversion of natural language data from clinical records and websites into formats that can be interpreted by computer systems. This is where ontologies play a significant role. The concept of ontologies refers to comprehensive frameworks that provide a structured representation of knowledge within a particular domain [8]. By defining a set of concepts and the relationships among them, ontologies facilitate the sharing and interoperable use of information across different systems. This capability is crucial for achieving semantic interoperability, ensuring that information exchanged between healthcare systems is understood in the same way by all parties.

Furthermore, ontologies contribute to the standardization of domain knowledge, promoting consistent practices across different platforms and applications. Unlike terminologies, which offer a system of terms for describing concepts within a domain, or classifications, which organize information into hierarchical categories, ontologies provide a richer semantic framework. They not only include a controlled vocabulary but also define the types of relationships that can exist between concepts, enabling a more comprehensive representation of domain knowledge. The development of ontologies aims to foster a common understanding of information structures among people or software agents [9]. Ontologies have been found to be an effective tool for capturing knowledge in databases and bioinformatics applications [10].

The purpose of this study was to develop a Fall Risk Management Ontology (FRMO) for various applications, including web semantics, nursing notes, and clinical data. We pursued three objectives as part of the FRMO development. The first objective was to identify concepts related to fall risk factors for fall risk assessments as it is essential to be aware of the factors that increase an individual’s risk of falling. The second objective was to identify the consequences of a fall as these consequences may result in future falls. The third objective was to identify fall prevention concepts to prevent individuals from falling again.

## Method

**Fig. 1:**
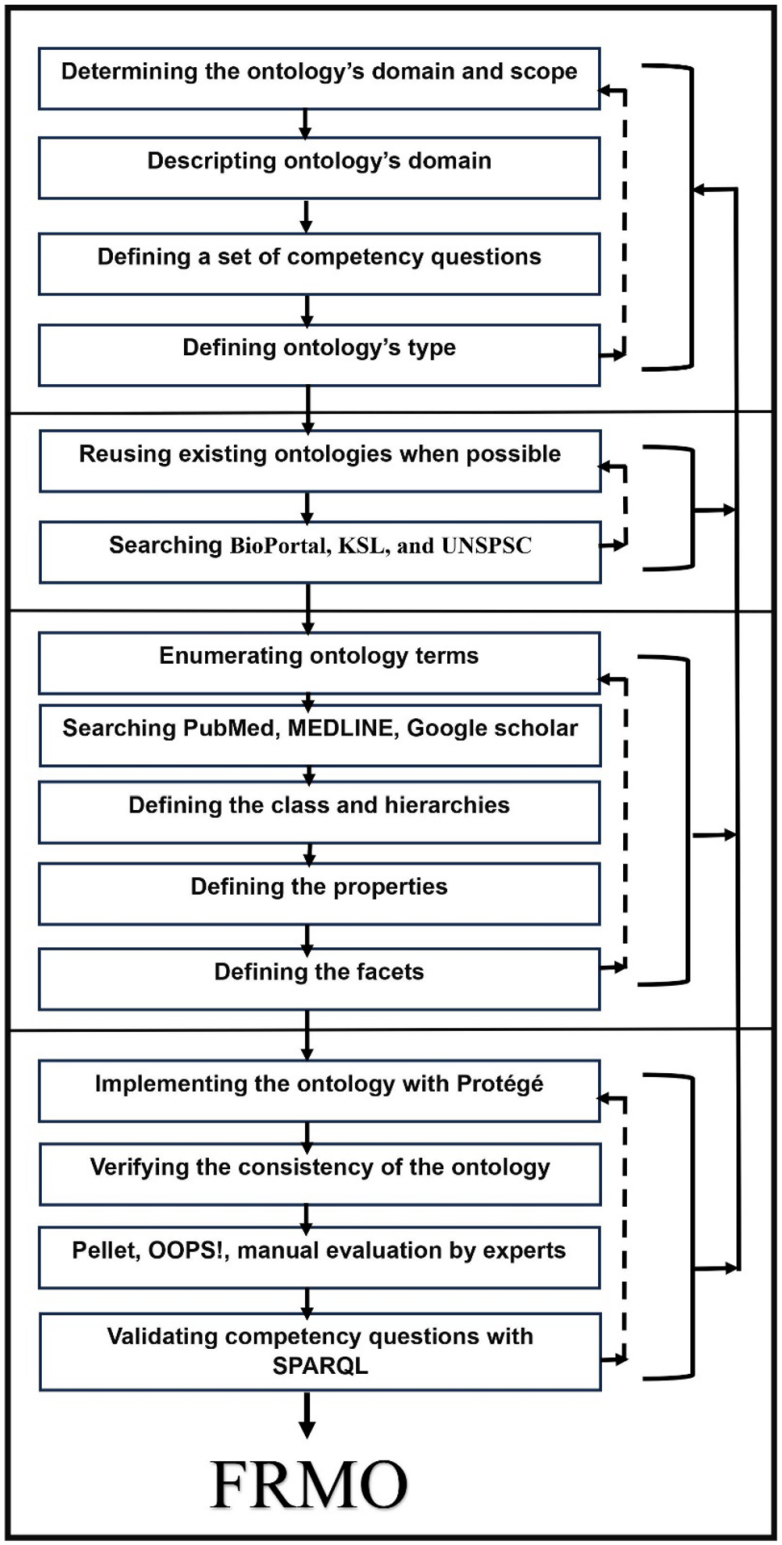
Development process of the FRMO

The development of the FRMO utilized the Ontology Web Language (OWL) within the Protégé ontology editor, a widely-adopted tool for building ontologies in various domains, including healthcare. Protégé, developed by Stanford University [11]. The ontology targets four types of users: front-end users, back-end users, researchers, and end users. Front-end users are developers or designers of interfaces to the semantic web containing information about fall risk factors, prevention, and consequences. Back-end users are software engineers who design systems using ontologies to help healthcare professionals manage fall risks; they also include researchers in the medical field working on studies aimed at improving older individuals’ quality of life. Finally, end users are healthcare professionals who work in residential care facilities or hospitals. Examples of use cases include managing fall risk as part of organizational improvements in residential aged care facilities and hospitals; exchanging fall risk information in real-time with people and machines to identify the risks’ causes and consequences; and developing data-driven strategies to identify people at risk of falling by linking various types of knowledge. Moreover, the FRMO can be utilized as background knowledge in an answering system based on ontologies for the interpretation of natural language questions. For the development of the FRMO, six of the Stanford approach’s seven steps were followed: (1) determining the ontology’s domain and scope, (2) reusing existing ontologies when possible, (3) enumerating ontology terms, (4) defining the classes and hierarchies of the classes, (5) defining the properties of the classes, and (6) defining the facets of the properties (see Fig. 1). The seventh step involves creating instances for the individuals in the ontology; in most cases, such instances are created by those who will use the ontology (e.g., hospitals or residential aged care facilities) [9]. Therefore, the seventh step was left to the end users.

In the step 2, several digital libraries of ontologies were searched to determine whether fall risk management ontologies had been developed before. In particular, three main ontology digital libraries were searched: BioPortal [12], KSL [13], and United Nations Standard Products and Services Code (UNSPSC) [14]. Three academic electronic libraries specializing in health-related research were searched for terms and concepts related to fall risk, fall risk factors, the assessment of fall risk, fall prevention, and fall consequences. These libraries included PubMed, MEDLINE, and Google Scholar (up to 4 January 2022). Moreover, the websites of the Victoria Government, the WHO, the New South Wales Government, and the Australian Government were searched (up to 4 January 2022) to gather additional information on fall risks.

In the step 3, a literature review was conducted to select the most relevant articles on fall risk. The titles and abstracts were screened in duplicate independently by two reviewers Following this, each reviewer independently identified appropriate articles. In case of discrepancies, the reviewers discussed their remarking on the articles. Articles, terms, and concepts were selected and critically assessed using the Systematic Reviews and Meta-Analyses (PRISMA) guidelines [15]. Articles were selected based on six inclusion criteria:

1. They were written in English and addressed fall risk;
2. They clearly identified fall risk factors;
3. They addressed fall prevention, risk, consequences, injuries, or characteristics;
4. The research types included descriptive studies, meta-analyses, case studies, systematic reviews, randomized controlled trials, and practical guidelines for fall risk management;
5. They were published from January 2000 to December 2021;
6. They were published in peer-reviewed scientific journals.

Articles were excluded based on the following exclusion criteria:

1. They failed to identify prevention terms, consequences, or risk factors associated with falls and
2. They were duplicate articles

In the step 4, to create classes and their hierarchy, a top-down approach, “which begins with defining broad concepts in the domain and then specializing the subsequent of the concepts”, was used [16]. The FRMO was evaluated based on four main evaluation concepts: consistency, completeness, accuracy, and clarity. We used four different evaluation approaches: (1) the reasoner Pellet; (2) OOPS!; (3) manual evaluation by experts; and (4) answering competence questions with SPARQL.

In the development of the FRMO using Protégé, a distinction was made between the steps automated by Protégé and those that required manual effort. Protégé, a widely-used ontology editor, offers automated tools for class creation, property assignment, and consistency checks to streamline the development process. These automated features were instrumental in maintaining the logical consistency of the FRMO and ensuring correct inferences regarding class hierarchies and relationships based on predefined rules and properties. However, the foundational tasks, such as defining the ontology’s domain and scope, identifying and detailing specific classes and their hierarchies, and determining the properties of these classes, necessitated a deep manual effort. This process involved comprehensive literature reviews and consultations with domain experts to accurately represent fall risk management knowledge. Among the domain experts who significantly contributed to the ontology was Eman Al Ribh, a nurse with practical experience in fall risk management. Her expertise was instrumental in validating the clinical relevance of the ontology, ensuring that the FRMO reflects real-world practices and needs within the healthcare setting.

## Results

The FRMO aims to provide a model representing knowledge in the domain of fall risk management. The scope of this ontology includes the identification of individuals at risk of falling and fall prevention strategies, as well as information about the consequences of a fall.

The step (1) was divided into two stages. First, the ontology’s domain was defined by answering questions such as “What is the ontology’s domain?,” “What is the purpose of the FRMO?,” and “Who will use it and maintain it?” Second, its scope was defined by defining a set of competency questions that an ontology-based knowledge base is expected to be able to answer. Competence questions can be used to express the requirements (expressiveness) for an ontology and to informally justify the need to develop the ontology [17]. Furthermore, the FRMO should provide answers to questions related to fall risk, such as “What factors cause falls?” Later, these questions were used as tests to determine whether the FRMO provides adequate information that can assist in fall management.

Upon step (2) searching the ontology digital search engines, The search did not yield any ontology related to fall risk management. However, some ontologies that have defined terms related to fall risk management have been integrated with the FRMO, such as the Human Disease Ontology (DOID), Symptom Ontology (SYMP), and Human Developmental Anatomy Ontology (TEMP). The search of the electronic libraries revealed several keywords related to the topic, including “fall”, “fall risk factors,” “fall risk assessment tool,” “fall prevention,” “fall risk management,” “fall risk scale,” “fall risk controlling,” “fall injuries”, “fall consequences,” “fall risk factors,” “fall and older people,” and “fall risk predicting.”

**Fig. 2:**
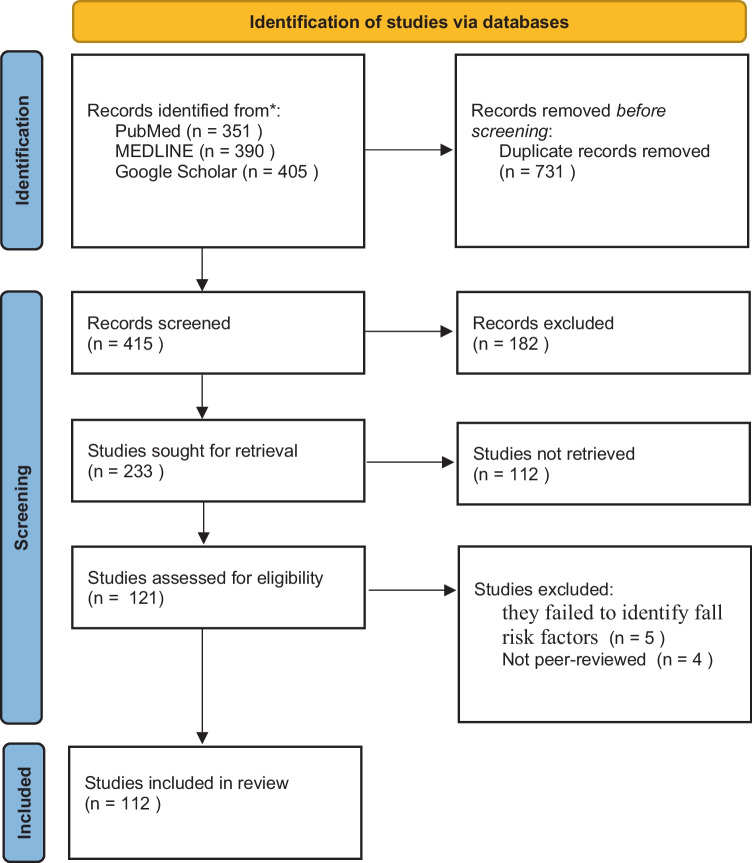
Flowchart of the modified PRISMA [15]

In step (3), the literature was reviewed to extract terms and concepts to achieve two aims. Our primary aim was to identify the key terms and concepts related to fall risk management. Our secondary aim was to provide a better understanding of those concepts. Significant concepts and terms related to fall risk management knowledge were extracted from the literature and coded in the ontology editor Protégé. The extracted nouns were divided into attributes, instances, and concepts. The main objective of the FRMO is to manage fall risk through concepts related to fall risk management, including fall risk factors for predicting falls before their occurrence; concepts and entities related to preventing falls; and the consequences of falls. After collecting all the relevant literature, we selected the most appropriate articles. Of the 1,146 articles collected, 112 articles remained after applying the inclusion and exclusion criteria (Fig. 2).

In step (4), the classes of the ontology were derived from the extracted nouns, while the hierarchy of the FRMO classes was based on the literature, with additional input from biomedical websites and services to provide more accuracy for the hierarchy of medicines and medical classes. To classify the medicines, first, the medicines leading to falls were identified. Then, the Drug Information Portal [18] was searched for their classifications and synonyms. To learn more about each drug, the search engine on the Therapeutic Goods Administration (TGA) page was used [19]; this search engine for medicines in Australia helped us to provide more information about the medicine and to classify it. The hierarchy of subclasses for the Medical class (Diagnoses) was determined through the Unified Medical Language System (UMLS) Metathesaurus Browser [20]. Each class contained annotations when applicable, such as a label, definition, synonyms, SNOMED_CT, and resources. A definition was added to classes whose purpose was unclear.

The FRMO consists of 890 classes arranged in a hierarchical structure, including six top-level classes. The six top-level classes are Consequences of Falls (an individual’s injuries and complications following a fall, including death), Fall Risk Assessment (fall risk assessments are used to determine if an individual is at low, moderate, or high risk of falling), Patients (any person who may suffer falls, be prone to falls, or be at risk of falling), Prevention (a fall prevention strategy is anything that can help prevent or reduce falls among vulnerable individuals), Risk Factors (risk factors for falls are parameters that increase an individual’s risk of falling), and Affected Body Parts (parts of the body that are injured when an individual falls).

A class hierarchy framework is not sufficient to address competency-related questions. Such questions require the definition of the relationships between the classes and between individuals. Step (5) The use of object properties enables classes to be related to other classes, and individuals to other individuals. For instance, the object property “has Risk Factors” connects the class “Patients” to the class “Risk Factors.” Ontologies also include data type properties, which describe the relationship between individuals and data types. As an illustration, “First Name” has the string data type. Determining the value type of the data type properties is known as defining the facets, which is the sixth step in the Stanford approach.

**Fig. 3:**
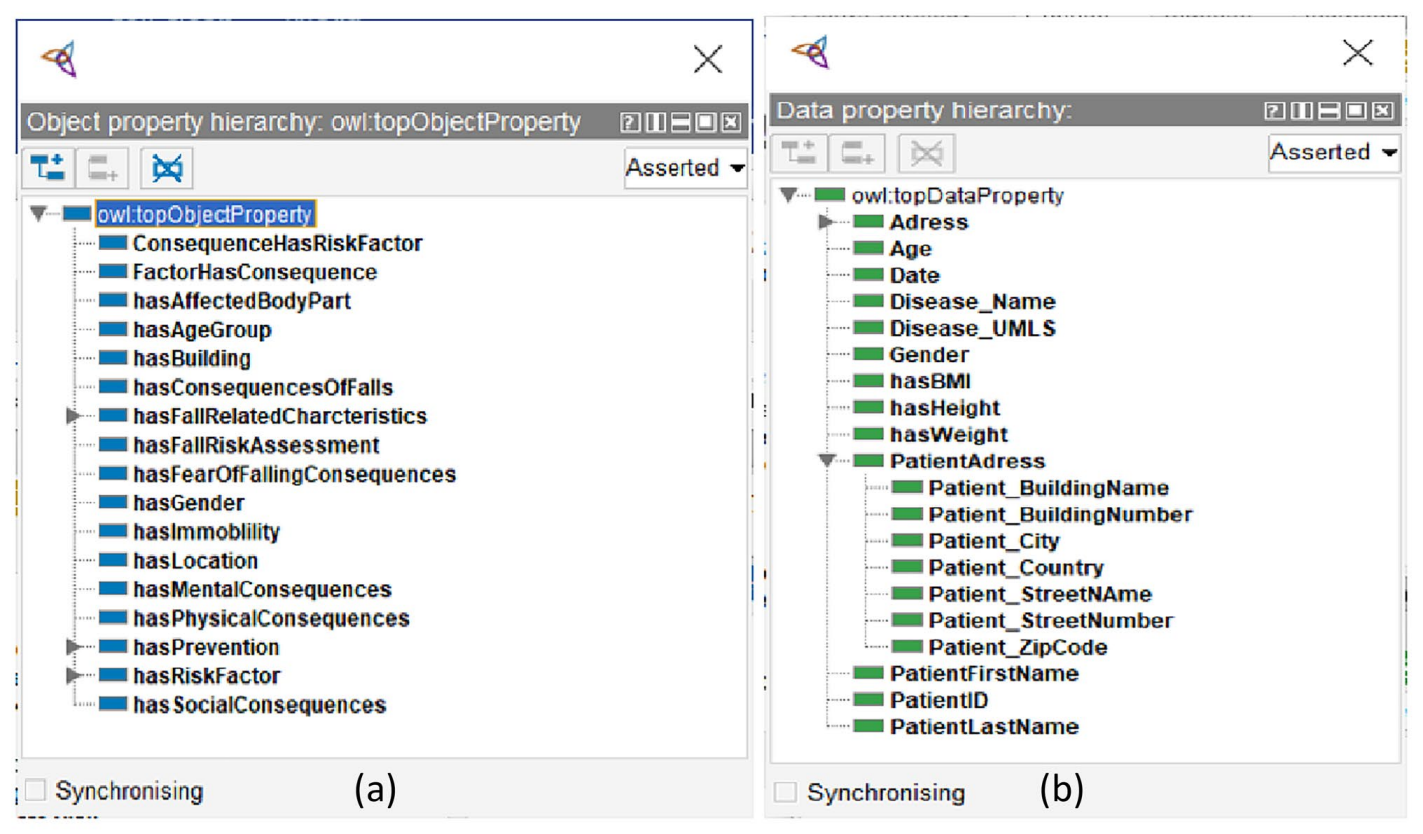
Screenshots of the FRMO within the Protégé ontology editor showing the hierarchy of **a** object properties and **b** data properties as defined in the ontology

Consistency among the classes and rules governing the ontology allows software to reason about the ontology [21]. The ontology’s completeness reflects how well a particular domain is covered [22]. In addition, an ontology is accurate when it describes classes, properties, and individuals correctly [22]. There are several subclasses for each top-level class, and each of these subclasses may further have its own subclasses. Furthermore, we developed a total of 43 object properties to link classes with domains and ranges. Some of these object properties are inverses of others. One example of a resultant object property is “hasFallRisk” and “hasPatients.” In this case, the property “hasFallRisk” links the individual “Patients” to the individual “RiskFactor.” For example, patient A has the risk factor “muscle weakness”. On the other hand, the category “muscle weakness” includes patient A. Moreover, “hasFallRisk” and “hasPatients” are inversely related (Fig. 3). In addition, we added 28 data properties to the FRMO to link values to certain classes, such as the data property ‘PatientFirstName,’ which is linked to the class ‘Patients’ (Fig. 3).

**Fig. 4:**
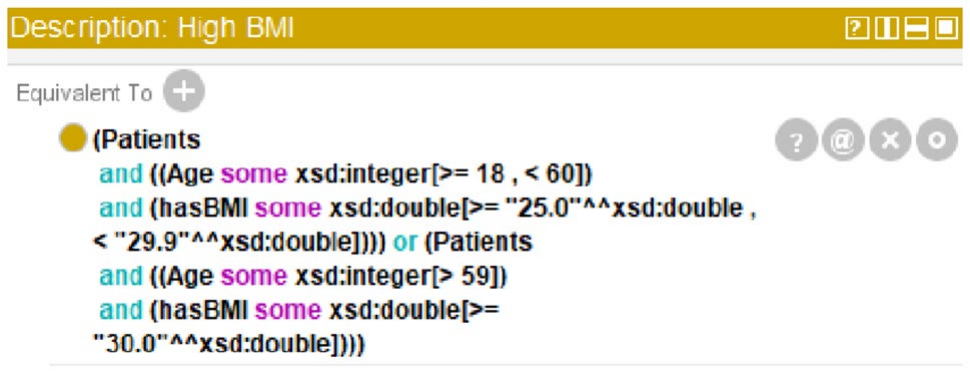
Screenshot from the Protégé ontology editor illustrating the logical conditions defining the ‘High BMI’ class within the FRMO

Clarity in an ontology means that it should convey the meaning of its defined classes and entities clearly [23]. As part of step (6), we added classes within the FRMO that correspond to BMI classes (High BMI, Normal BMI, Low BMI) and age groups (e.g., 100 age group). To clarify, the High BMI class is equivalent to the Patient class and the score for the High BMI class (Fig. 4) [24, 25].

Pellet, the first OWL 2 reasoner that comes with the Protégé editor, was used during the process of implementation to verify and evaluate the consistency of the relationships between classes, subclasses, and instances, as well as the class–instance relationships. Pellet allows ontology engineers to debug ontologies, evaluate instances, and define data types. In addition, it provides a range of features such as query response, rule reasoning support, axiom formulation, and model validation [26]. Pellet’s final results showed that the FRMO is consistent, indicating that every class, object property, and data property in the ontology has been assigned to its correct class and follows the correct rules. The reasoner also displayed the inferred classes, properties, and data properties, if any, following the rules of the FRMO. For example, Pellet inferred patients’ BMI subclasses and age groups when they were entered into the ontology as individuals.

For further evaluation, we used OntOlogy Pitfall Scanner! (OOPS! ), which is a web application that assists in identifying ontology development errors [27]. OOPS! detects common pitfalls in ontology development, such as wrong inferences and issues related to ontology clarity and real-world modeling. OOPS! checks a total of 41 ontology pitfalls in two categories: dimensions and evaluation criteria. Dimensions include structural dimensions (wrong inference, no inference, ontology language, and modeling decisions), functional dimensions (real-world modeling or common sense, completeness of requirements, and application context), and usability-profiling dimensions (ontology understanding, ontology clarity, and ontology metadata). The evaluation criteria category includes consistency, completeness, and conciseness [27].

**Fig. 5:**
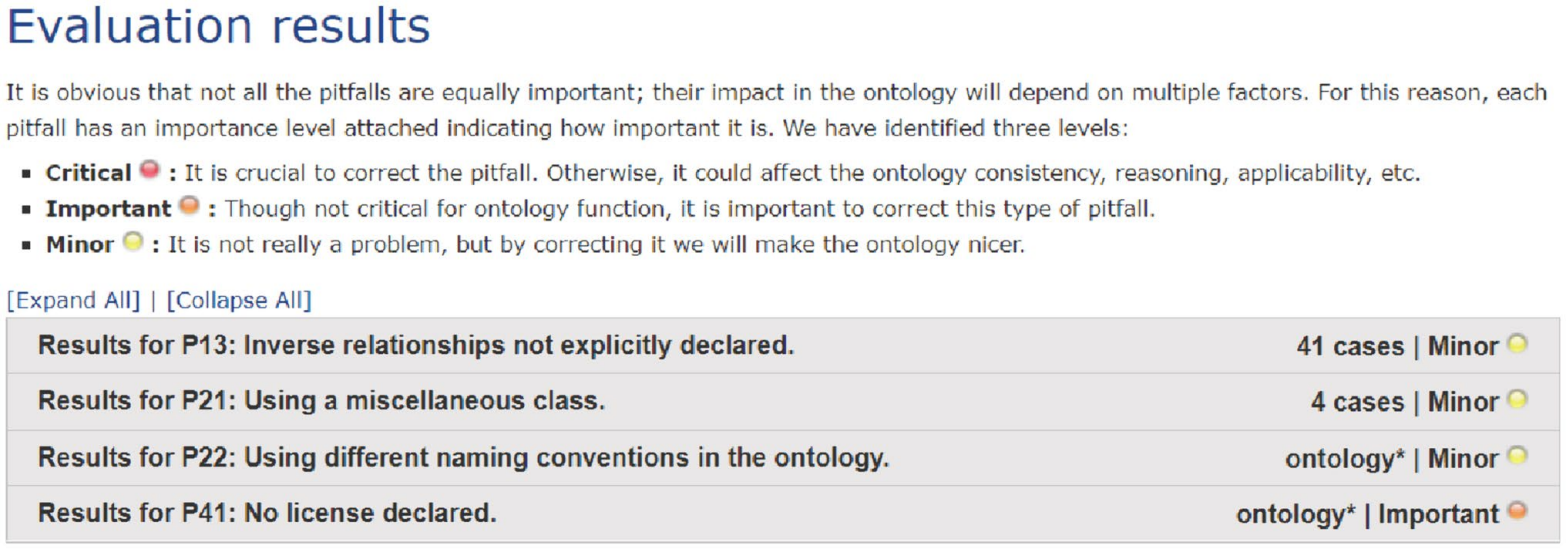
OOPS! evaluation for FRMO

The OOPS! framework classifies pitfalls into three categories: minor, important, and critical. There were three minor pitfalls and one important pitfall in the FRMO. The first minor pitfall was that 41 object properties did not have inverses. In ontologies, however, not all object properties must have inverse functionality; therefore, this pitfall is not a problem (Fig. 5). The second minor pitfall occurred when the word “other” was used (e.g., OtherLocation, Other_Shoe_Style, and Other_Direction_of_Fall). The word “other” was used in case any related information was not included in the ontology. The third minor pitfall was that the name of the ontology elements differed from the naming convention as some classes’ names did not include “_”; however, this will not affect the ontology (Fig. 5). The important pitfall was that the ontology metadata failed to include the license information for the FRMO. To prevent license conflicts, ontologies should be made public, meaning that they should be released without restrictions. This issue was solved by adding a Creative Commons license (http://creativecommons.org/license/zero) in the ontology header [28]. We created a new annotation property, “dc: rights,” and then used it to include the license in an ontology header.

We conducted a manual evaluation of the FRMO to assess its completeness, accuracy, and clarity. Following feedback on completeness from an expert nurse, we expanded the ontology to include two new terms. We added the “Vertigo” class as a subclass of “Dizziness” and the “Unexpected_Death” class as a subclass of “Consequences of Falls.” The expert nurse, with profound knowledge of fall risk factors, prevention strategies, and consequences within the field of fall risk management, provided crucial insights in affirming the completeness and applicability of the ontology’s terminology. As she was not a member of the development team, her assessment offered an unbiased perspective on the ontology’s accuracy and clarity.

As part of the FRMO, 869 terms related to fall risk management are used, including 39 terms related to affected body parts, 81 terms related to fall consequences, 68 terms describing fall characteristics, 4 terms defining fall risk assessment, 8 terms related to patients, 46 terms referring to prevention, and 623 terms related to risk factors. The study [29] provides general information about risk factors such as muscle weakness, whereas the FRMO provides more specific terms related to muscle weakness, such as leg weakness [30]. In addition, [29] targeted inpatients older than 64 years of age, while the FRMO targets patients older than 44 years regardless of whether they are inpatients or not.

Thereafter, we confirmed the accuracy of the FRMO. Finally, the FRMO was assessed by evaluating its ability to answer the competence questions. The competence questions were represented as queries using SPARQL to extract data from the FRMO. Several SPARQL requests were applied to the FRMO, such as “List all environmental risk factors among the extrinsic risk factors”, “List all risk factors for falls affecting the head in the 70 age group”, and “List the history of falls of patients associated with Rheumatoid Arthritis”. The SPARQL codes can be found online (https://github.com/ftuhaifa/SPARQL-for-FARMO). SPARQL for Competence question 1 “What are the factors that cause falls?” was as follows in Algorithm 1:

**Algorithm 1:** SPARQL for competence question 1

1: PREFIX rdf: <http://www.w3.org/1999/02/22-rdf-syntax-ns#>
2: PREFIX owl: <http://www.w3.org/2002/07/owl#>
3: PREFIX rdfs: <http://www.w3.org/2000/01/rdf-schema#>
4: PREFIX xsd: <http://www.w3.org/2001/XMLSchema#>
5: PREFIX: <http://www.semanticweb.org/ftuha/ontologies/2021/5/FRMO-01/>
6: SELECT? ind
7: WHERE { ?obj rdfs: subClassOf <http://www.semanticweb.org/ftuha/ontologies/2021/5/untitled-ontology-175/RiskFactors>. ?ind rdfs: subClassOf? obj}

We found that the FRMO produced the correct answer for each competence question based on the correlation between the original data and extracted data. The final version of the developed FRMO can be found online (https://bioportal.bioontology.org/ontologies/FRMO).

## Discussion

**Fig. 6:**
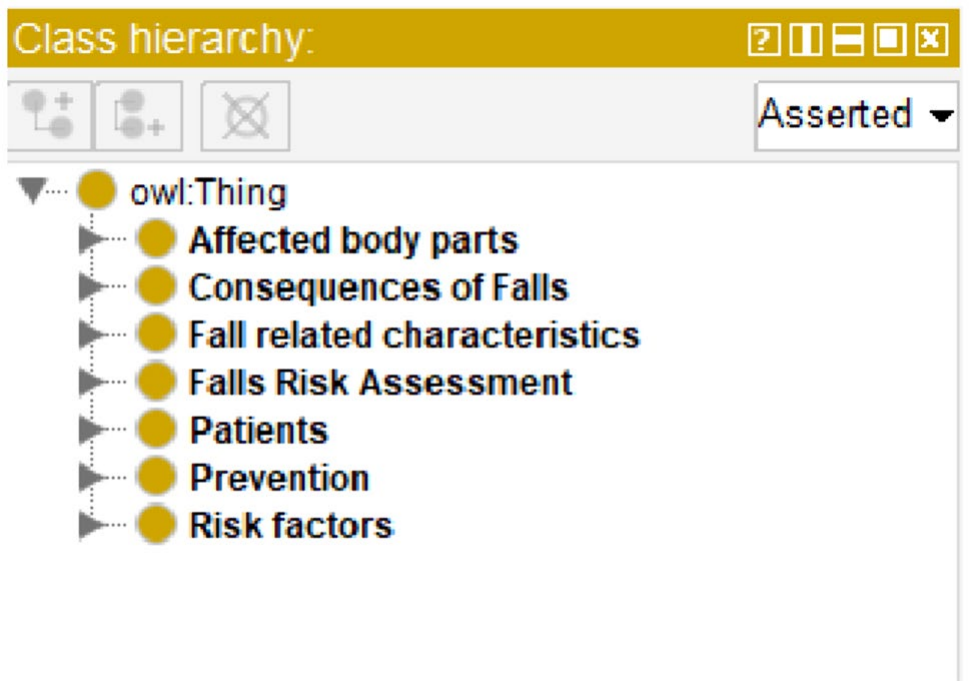
Class hierarchy in the FRMO as visualized in the Protégé ontology editor, displaying the top-level classes that encompass the domain of fall risk management, including ‘Affected body parts,’ ‘Consequences of Falls,’ ‘Fall

In reviewing the development of the FRMO, we reflect on our initial objectives: to identify concepts for fall risk factors, consequences, and prevention. The screenshot from the ontology (Fig. 6) demonstrates a hierarchical organization of classes that directly correspond to these aims.

1. **Fall Risk Factors**: We successfully enumerated terms related to risk factors, which are essential for predicting falls before their occurrence. The inclusion of 623 terms pertaining to risk factors indicates a thorough coverage of this aspect.
2. **Consequences of Falls**: Our ontology now includes 81 terms related to fall consequences, highlighting the breadth of potential outcomes following falls. This variety supports healthcare providers in understanding and addressing the aftermath of falls.
3. **Prevention**: With 46 terms dedicated to prevention, the FRMO provides valuable insights into proactive measures. This supports the development of targeted interventions to reduce the incidence of falls.

The classes for ‘Affected body parts’ and ‘Fall related characteristics’ also contribute to a richer understanding of fall incidents, which aids in comprehensive risk assessments and the formulation of detailed care plans. Through these organized classes and terms, the FRMO offers a robust framework for fall risk management, adhering to the initial objectives set forth in the study. The ontology’s structure allows for detailed data analysis, from identifying risk factors to informing prevention strategies. The objectives of identifying key concepts for fall risk, consequences, and prevention in the FRMO could potentially have been achieved using alternative methods, such as machine learning algorithms for text mining or leveraging existing healthcare databases. However, these alternatives might not provide the same depth of semantic relationships or ease of integration with clinical decision systems as the ontology-based approach adopted in this study.

By developing an ontology, domain scientists share a common language and understanding of the subject matter. The FRMO will allow scientists to share knowledge about falls and extract relevant data from natural language texts and clinical data. Developing an ontology remains useful to support fall prediction from natural language texts and clinical data, to facilitate fall prevention programs, and to identify the consequences of falls in order to treat them. Thus, the FRMO is intended to allow domain scientists to share a common language about fall risk management. By studying the literature on fall risk management, we acquired knowledge regarding fall risk factors, interventions, and consequences that was helpful in determining the ontology’s required rules and concepts. In contrast to other ontologies, the FRMO encompasses a rich set of concepts about fall risk factors that can be used to determine whether an individual is at risk of falling. Based on the determined fall risk factors, a prevention program can be developed. Furthermore, the FRMO includes a rich set of concepts associated with falls and their consequences. To assist in linking datasets within a related domain, each concept, object property, and data property is annotated with a label [31]. The FRMO also contains synonyms for every concept, allowing it to assist in natural language processing (NLP) [9]. NLP is an essential tool for extracting meaningful information from unstructured data sources such as clinical notes or research articles. The FRMO facilitates this process by serving as a semantic foundation upon which NLP algorithms can operate. It allows for the translation of natural language into structured data that can be more easily analyzed and processed, enabling the development of advanced clinical decision support systems.

As information sources contain a wide range of structured and unstructured information, it has become more important to develop efficient methods of retrieving information [32]. To date, there are several methods of automated information retrieval, such as Query By Example (QBE), Query By Template (QBT), and Universal Interface with Conceptual Knowledge (UICK) [16]. These methods, however, cannot support complex query formulation or semantic retrieval [16]. Furthermore, some approaches cannot provide reliable results because they lack domain knowledge, which can lead to inaccuracies. The ontology approach can overcome these shortcomings [16]; indeed, the FRMO can handle these issues and generate intelligent queries for relational databases. The use of different methods resulted in a comprehensive and effective evaluation. Moreover, the process of testing the FRMO’s efficacy, utilizing competence questions through SPARQL queries, underscores the ontology’s potential in structured scenarios. While these tests are instrumental in demonstrating the FRMO’s capabilities in knowledge representation and query processing, they also pave the way for further advancements. The evolution of the FRMO, through iterative development and feedback, promises to refine its structure and content, aligning it more closely with the dynamic needs of healthcare professionals in fall risk management.

While the FRMO has been developed with a high degree of accuracy in terms of its content and structure, limitations arise from the fact that this ontology is based on English language and SNOMED CT, which means it may have limitations in regions that do not speak English or use SNOMED CT. For organizations utilizing SNOMED CT, language barriers are mitigated as the FRMO’s integration with SNOMED CT enhances interoperability and aligns with global medical terminologies. SNOMED CT’s expansive clinical vocabulary supports consistent data management across healthcare sectors, regardless of language. This standardization allows the FRMO to effectively interface with diverse systems and participate in a unified healthcare data framework, essential for its widespread application in varying healthcare environments.

## Conclusion

This article presents the FRMO, which represents the domain knowledge within fall risk management for people between the ages of 40 and 100 years. Our search highlighted a gap in ontologies for clinical fall risk management, leading to the creation of the FRMO based on best evidence and the adapted Stanford approach. As a result of the FRMO, various applications can be developed, particularly in the field of clinical decision-making technologies that assist healthcare providers in managing falls among older adults. The site provides a valuable resource for the design of user interfaces, the creation of healthcare systems by back-end developers, the study of the quality of life of the elderly by researchers, and the provision of care by healthcare professionals. In the future, the FRMO will be integral to systems with user interfaces that clinicians interact with, facilitating the integration of semantic web information into everyday clinical practice. The next phase will involve connecting this system to data sources using Owlready2 [33], a Python module that enables ontology-oriented programming and supports data linkage. This progression will provide healthcare professionals with actionable insights, derived from a rich network of linked data, to inform their decisions and strategies for fall prevention and management.

## Data Availability

This study did not use or analyze the datasets. However, the ontology can be found at (https://bioportal.bioontology.org/ontologies/FRMO/?).
